# Delirium as a predictor of sepsis in post-coronary artery bypass grafting patients: a retrospective cohort study

**DOI:** 10.1186/cc9273

**Published:** 2010-09-27

**Authors:** Billie-Jean Martin, Karen J Buth, Rakesh C Arora, Roger JF Baskett

**Affiliations:** 1 Department of Cardiac Sciences, University of Calgary, 8th Floor Cardiology, 1403 29th Street NW, Calgary, Alberta T2N-2T9, Canada; 2 Division of Cardiac Surgery, Department of Surgery, Dalhousie University, 2269-1796 Summer Street, Halifax, Nova Scotia B3H 3A7, Canada; 3 Sections of Cardiac Surgery and Critical Care, Department of Surgery, University of Manitoba, CR3012 - 369 Tache Ave, St. Boniface General Hospital/I.H. Asper Clinical Research Institute, Winnipeg, MB R2H2A6, Canada

## Abstract

**Introduction:**

Delirium is the most common neurological complication following cardiac surgery. Much research has focused on potential causes of delirium; however, the sequelae of delirium have not been well investigated. The objective of this study was to investigate the relationship between delirium and sepsis post coronary artery bypass grafting (CABG) and to determine if delirium is a predictor of sepsis.

**Methods:**

Peri-operative data were collected prospectively on all patients. Subjects were identified as having agitated delirium if they experienced a short-term mental disturbance marked by confusion, illusions and cerebral excitement. Patient characteristics were compared between those who became delirious and those who did not. The primary outcome of interest was post-operative sepsis. The association of delirium with sepsis was assessed by logistic regression, adjusting for differences in age, acuity, and co-morbidities.

**Results:**

Among 14,301 patients, 981 became delirious and 227 developed sepsis post-operatively. Rates of delirium increased over the years of the study from 4.8 to 8.0% (*P *= 0.0003). A total of 70 patients of the 227 with sepsis, were delirious. In 30.8% of patients delirium preceded the development of overt sepsis by at least 48 hours. Multivariate analysis identified several factors associated with sepsis, (receiver operating characteristic (ROC) 79.3%): delirium (odds ratio (OR) 2.3, 95% confidence interval (CI) 1.6 to 3.4), emergent surgery (OR 3.3, CI 2.2 to 5.1), age (OR 1.2, CI 1.0 to 1.3), pre-operative length of stay (LOS) more than seven days (OR 1.6, CI 1.1 to 2.3), pre-operative renal insufficiency (OR 1.9, CI 1.2 to 2.9) and complex coronary disease (OR 3.1, CI 1.8 to 5.3).

**Conclusions:**

These data demonstrate an association between delirium and post-operative sepsis in the CABG population. Delirium emerged as an independent predictor of sepsis, along with traditional risk factors including age, pre-operative renal failure and peripheral vascular disease. Given the advancing age and increasing rates of delirium in the CABG population, the prevention and management of delirium need to be addressed.

## Introduction

Cardiac surgery is increasingly being performed on older patients with limited physiologic reserve and multiple medical co-morbidities [1]. A significant number of patients, especially the elderly, develop peri-operative neurological complications ranging from subtle cognitive dysfunction and mild confusion to frank delirium, and occasionally permanent stroke. The prevalence of delirium after cardiac surgery has been reported to be as low as 3%, and as high as 72% [2-4].

The importance of delirium is frequently dismissed, as it is seen as a transient entity. It is, however, the most common neurological complication after cardiac surgery [5]. Multiple pre-operative predictors of delirium have been uncovered including advanced age, previous stroke, and various medications [5]. Post-operative delirium can be very difficult to manage once it has occurred. The efficacy of delirium treatment strategies published thus far are at best modest [6].

Delirium after cardiac surgery has been shown to increase hospital and ICU stay, and may even be life threatening [5]. Furthermore, long-term survival and quality of life have been shown to be adversely effected in those who suffer peri-operative delirium [7]. However, there are many other outcomes of interest that have not been investigated as they relate to delirium. In particular, while it is known that delirium is a common sign of end organ dysfunction in sepsis, there is no published literature examining the relationship between delirium preceding infectious complications, including sternal wound infection, pneumonia, urinary tract infections, and sepsis. As delirious patients are difficult to properly care for and frequently exhibit behaviors that may predispose them to infection such as not following sternal precautions, failing to clear secretions, and requiring catheters for long periods, the authors suspect that delirious patients may be more likely to develop sepsis. The objective of this study therefore was to determine if preceding delirium is associated with sepsis following CABG surgery, or simply a consequence.

## Materials and methods

### Patient population

This study included all patients undergoing isolated CABG surgery at the Queen Elizabeth II (QEII) Health Sciences Centre in Halifax, Nova Scotia, Canada, and in two cardiac centers in Winnipeg, Manitoba, Canada between June 1998 and July 2007. The QEII Health Sciences Centre is the sole cardiac surgical center in the province of Nova Scotia as well as parts of surrounding provinces. The Health Sciences Center and St. Boniface General Hospital are the only cardiac surgical centers serving the province of Manitoba.

### **Data collection and variable selection**

The Maritime Heart Center Cardiac Surgery Registry and the Manitoba Cardiac Surgery Database are detailed clinical databases that prospectively capture pre-, intra-, and post-operative information on all cardiac surgery patients. The Manitoba Heart Database captures data from both centers in the province that conduct heart surgery. The two databases include cases from the same time period and were created using the same Society of Thoracic Surgeons (STS) data definitions, allowing them to be concatenated. Delirium was defined as per the STS definition as, "mental disturbance marked by illness, confusion, cerebral excitement, and having a comparatively short course" [8].

Preoperative characteristics included age, sex, smoking history, body mass index (BMI, kg/m^2)^, hypertension, diabetes, hypercholesterolemia, chronic obstructive pulmonary disease (COPD), congestive heart failure (CHF), pre-operative length of stay (LOS), recent myocardial infarction (MI) (occurring within 21 days prior to surgery), pre-operative renal insufficiency (RF, Cr >176 μmol/L), peripheral vascular disease (PVD), cerebrovascular disease (CVD), ejection fraction (EF) <40%, urgency (emergent surgery defined as occurring in the next available operating time; these patients have ongoing, cardiac compromise and are unresponsive to any therapy except cardiac surgery) and redo cardiac surgery. The primary outcome of interest was sepsis. Sepsis was defined as "post-operative clinical syndrome of sepsis, with positive blood cultures" [8]. Additionally, we included 22 patients as septic who clinically met the criteria for Systemic Inflammatory Response Syndrome (SIRS) but who did not have positive blood cultures, but either (a) had these cultures drawn after the initiation of antibiotics, and/or (b) had other positive cultures (sputum, sternum, urine). In septic patients who did not have positive blood cultures, the onset of sepsis was determined by the timing of the first diagnosis of sepsis or SIRS in physician charting. Patients were screened for sepsis over the entire course of their hospitalization.

A retrospective review of the charts of all septic patients were undertaken to determine the time between onset of delirium and sepsis. Patients were considered to be delirious first only if delirium preceded sepsis by a minimum of 48 hours, with no clinical signs of sepsis between the onset of delirium and time of drawing of blood culture. Other data collected on chart review included identification of microbe grown in the blood cultures of the septic patients.

Full ethics approval was obtained from all three institutional research ethics boards, in keeping with the Tri-Council Policy Statement: Ethical Conduct for Research Involving Humans. A waiver of informed consent was granted by all three research ethics boards as the study did not involve therapeutic interventions or potential risks to the involved subjects.

### Statistical analysis

All analysis was done on the combined group of patients from the two databases. Prior to concatenating the databases, rates of delirium and sepsis were compared between the two using chi-squared tests to ensure they were comparable. Univariate comparisons of pre-operative characteristics between delirious and non-delirious patients, and between patients who developed sepsis and those who did not, were conducted using χ^2 ^tests or Fisher's exact tests for categorical variables.

The association between delirium (defined as delirium that preceded sepsis by at least 48 hours) and sepsis was assessed by logistic regression after adjusting for relevant risk factors. Clinical variables with univariate chi-square *P *< 0.20 were presented to the model; by backward elimination only variables significant at *P *≤ 0.05 were retained. A receiver operating characteristic (ROC) curve was calculated as a measure of sensitivity and specificity for the logistic regression model. A bootstrap procedure was performed on 200 subsamples to confirm the independent predictors of sepsis; furthermore, the 95% confidence interval of the ROC was obtained from the 2.5th and 97.5th percentiles of the bootstrap distribution. Statistical analysis was performed using SAS software version 9.1 (SAS, Cary, NC, USA).

The authors had full access to the data and take full responsibility for its integrity. All authors have read and agree to the manuscript as written.

## Results

**Figure 1:**
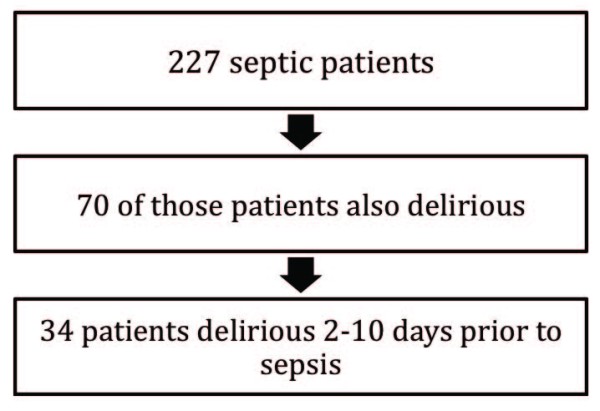
**Flowchart of septic patients by presence and timing of delirium**.

Between June 1998 and July 2007, a total of 14,301 patients underwent isolated CABG surgery at the QEII Health Sciences Centre and the two Winnipeg centers. Thirty-nine patients were eliminated from the analysis due to missing data. Of the remaining 14,262 patients, 981 (6.9%) developed delirium, and 227 patients (1.6%) developed sepsis. Of the septic patients, 70 also had delirium, 34 of whom clearly developed delirium between 2 and 10 days prior to the onset of sepsis (Figure 1). Rates of delirium and sepsis were compared between the two provinces. Rates of sepsis were higher in Nova Scotia than Manitoba (2.32% versus 0.85%, *P *< 0.001), but rates of delirium were equivalent (*P *= 0.32), as were rates of delirium in the septic patients (*P *= 0.06).

Those patients that developed post-operative delirium were more likely to have diabetes, renal insufficiency, COPD, PVD or CVD, and pre-operative atrial fibrillation (all *P *< 0.0001) (Table 1). Furthermore, they were more likely to have undergone a redo or emergent procedure (*P *< 0.0001). The patients who became delirious were more likely to develop pneumonia, urinary tract infections, deep sternal wound infections, and sepsis (all *P *< 0.0001) (Table 2). In addition, patients who became septic had a greater pre-operative length of stay than those who did not (Table 3). In those patients who did become septic, the mean time between their operation and diagnosis of sepsis was 10.52 days (standard deviation (SD) 13.97 days). The mean length of stay in ICU prior to the diagnosis of sepsis was 3.53 days (SD 8.16 days). However, not all patients were in ICU at the time of development of their delirium or sepsis.

**Table 1:** Pre-operative patient characteristics by delirium

	Delirium	No Delirium	*P*-value
Pre-operative length of stay more than seven days	17.4	15.4	0.087
Male	78.0	76.4	0.223
Age ≥70 (%)	59.2	33.6	<0.0001
Diabetic (%)	43.5	34.2	<0.0001
Renal Insufficiency (%)	7.4	4.0	<0.0001
COPD (%)	23.1	14.7	<0.0001
PVD/CVD (%)	43.2	25.6	<0.0001
Emergent Surgery (%)	8.0	3.8	<0.0001
Stroke (permanent) (%)	5.6	1.5	<0.0001
BMI ≥35	8.3	11.4	0.0025
3v/LM disease	86.3	78.0	<0.0001
Hypertension	75.0	66.5	<0.0001
Current smoking	18.8	16.8	0.10
Pre-operative atrial fibrillation (%)	10.9	6.1	<0.0001
Redo Surgery	5.1	3.3	0.0032
Ejection Fraction <40% (%)	23.1	14.7	<0.0001
Sepsis	7.4	1.2	<0.001

COPD, Chronic Obstructive Pulmonary Disease; PVD/CVD, Peripheral Vascular Disease/Cerebrovascular Disease; 3v/LM disease, Triple Vessel/Left Main disease.

**Table 2:** Infectious complications by delirium

	Delirium	No Delirium	*P*-value
Pneumonia (%)	20.35	3.80	<0.0001
Urinary Tract Infections (%)	13.8	3.0	<0.0001
Deep Sternal Wound Infection (%)	1.93	0.44	<0.0001
Sepsis (%)	7.43	1.18	<0.0001

**Table 3:** Pre-operative patient characteristics by sepsis

	Sepsis	No Sepsis	*P*-value
Pre-op length of stay more than or equal to seven days	29.3	15.3	<0.0001
Male (%)	70.9	76.6	0.044
Age ≥70 (%)	51.6	35.1	<0.0001
Diabetic (%)	45.7	34.9	0.0007
Renal insufficiency (%)	14.8	3.9	<0.0001
COPD (%)	23.1	14.7	<0.0001
PVD/CVD (%)	44.8	26.6	<0.0001
Emergent surgery (%)	13.9	3.9	<0.0001
Stroke (permanent) (%)	11.4	1.7	<0.0001
BMI ≥35	14.9	11.2	0.080
3v/LM disease	94.4	78.3	<0.0001
Hypertension	73.0	67.0	0.053
Current smoking	18.7	16.9	0.47
Pre-operative atrial fibrillation	15.7	6.3	<0.0001
Redo surgery	7.4	3.4	0.0009
Ejection fraction <40% (%)	36.1	14.9	<0.0001
Delirium	28.9	6.5	<0.0001

COPD, Chronic Obstructive Pulmonary Disease; PVD/CVD, Peripheral Vascular Disease/Cerebrovascular Disease; 3v/LM disease, Triple Vessel/Left Main disease.

Septic patients had higher rates of diabetes, CVD, renal insufficiency and COPD (all *P *< 0.0001). In addition, they were older, more likely to be delirious, and more likely to have stayed in hospital for more than seven days prior to undergoing surgery (all *P *< 0.0001).

The causative organism was identified in the large majority of septic patients (Table 4). Over half of the patients grew *Staphylococci*, with two cases of Methicillin Resistant *Staphylococcus aureus *infection. More than two causative organisms were identified in 8.8% of patients. No organism was identified in 9.7% of patients.

**Table 4:** Responsible organism in blood cultures

	Percent of patients (*n *= 227)
Staphylococcal	51.1
Staphylococcus	26.0
Coagulase	24.2
Methicillin	0.9
Enterococcus	7.0
Klebsiella	6.6
Enterobacter	4.0
Pseudomonas	3.1
Streptococcal	1.3
Candida	0.9
Other	7.5
Two or more organisms	8.8
No organism identified	9.7

A multivariate analysis was performed with a focus on patients who were deemed delirious for more than 48 hours prior to a diagnosis of sepsis. In these patients, delirium was significantly associated with post-operative sepsis with an OR of 2.32 (95% CI 1.59 to 3.39) after adjusting for pre-operative prognostic variables (Table 5). Other variables associated with sepsis included the pre-morbid conditions of elevated BMI, CHF, PVD/CVD, renal insufficiency, and atrial fibrillation (all *P *< 0.05). Emergent surgery, redo operation, and a pre-operative in-hospital stay of more than seven days were also associated with sepsis. The ROC for the sepsis model was 77.2%, 95% CI 76.6 to 82.5.

**Table 5:** Risk-adjusted impact of delirium on sepsis

	OR	95% CI
Delirium	2.32	1.59, 3.39
Age squared	1.17	1.04, 1.32
BMI ≥35	1.87	1.25, 2.78
CHF	1.79	1.28, 2.50
PVD/CVD	1.40	1.03, 1.89
Renal Insufficiency	1.85	1.17, 2.93
Pre-op LOS more than seven days	1.61	1.14, 2.26
Emergent OR	3.32	2.17, 5.10
Redo operation	1.89	1.10, 3.22

ROC 77.2%, 95% CI 76.6 to 82.5.

OR indicates odds ratio, CI indicates confidence interval.

## Discussion

This represents the largest analysis of delirium in the cardiac surgical population published to date. In this study of over 14,000 isolated CABG patients, we have confirmed that delirium is prevalent post-operatively, and have found evidence to suggest an association between delirium and sepsis.

It has been widely recognized that delirium can be a symptom of end organ dysfunction in sepsis. However, this is the first analysis to suggest that delirium may in fact play a role in the development of sepsis. Importantly, in this study cohort, delirium was found to precede the overt diagnosis of sepsis in 30.8% of patients, thus suggesting that delirium may put patients at increased risk of developing sepsis.

Delirium is common in the general ICU population with an estimated prevalence of up to 62% [9] and common in the post-operative cardiac surgical population [5]. There have been many models developed to predict its development. In the general ICU population, delirium has been associated with prolonged ventilation times, self-extubation, and re-intubation [10]. Prolonged mechanical ventilation, as well as an increased number of airway procedures, are known to increase the risk of nosocomial pneumonia and the subsequent development of sepsis [11]. Delirium has also been associated with removal of catheters, resulting in increased instrumentation of the urinary tract and increased risk of the development of urinary tract infections [12]. Furthermore, there is a pharmacological armamentarium used to treat delirium, including anticholinergic medications that have side effects including decreased secretions and urinary retention, and a number of sedating agents that decrease the amount of time patients are likely to spend ambulatory and may impact their ability for self-care.

Delirious patients have been shown to have poor oral intake and are at increased risk of developing malnutrition [13]. Malnutrition significantly impairs immune function, putting the patient at increased risk of peri-operative infectious complications [14]. Furthermore, delirious patients experience a loss of day-night orientation, have significant disruption to regular sleep patterns and are frequently sleep deprived [15]. A number of immunological functions are dependent on circadian rhythms and regular sleep, and those who are sleep deprived are therefore less able to mount an appropriate immune response to pathogens [16].

Patients with delirium often have prolonged intubation times [5], are less likely to comply with sternal precautions, require prolonged bladder catheterization, and are less likely to mobilize [12,17]. Furthermore, owing to poor nutrition [18], disruption to their sleep-wake cycle [15], and disruptions in their natural defenses [19], patients with delirium may be more prone to develop sepsis with any given infection. In lieu of these features, we hypothesized that delirium may precede infectious complications, and sepsis.

A number of other findings in our study warrant mention. In particular, pre-operative atrial fibrillation was found to be associated with delirium and sepsis, both univariately and in the multivariate analysis. This is likely due to the fact that atrial fibrillation is indicative of global physiologic impairment, rather than being causative of either entity [20].

Despite our attempts to clearly delineate a timeline between the onset of delirium and sepsis, there remains the possibility that delirium may in fact be an early marker of sepsis rather than a predictor. Allowing that to be the case, the findings of this study can still be considered to be of merit: at the very least, perhaps delirium should be thought of as a prompt to expeditiously investigate for sepsis.

There are several limitations that should be noted. This is a three centre, retrospective study with the inherent bias and confounding in such studies. Furthermore, our analysis was limited by the STS definition of delirium, which is strictly that of an agitated delirium. As such, it is likely that a significant number of patients with hypoactive delirium or sub-syndromal delirium were not classified as delirious in this study. There was not an *a priori *protocol for delirium management at any of the study sites; each institution treated delirious patients as per the discretion of the attending physician. Both of these issues could be addressed in quality improvement projects which: (1) identify delirious patients based on a more inclusive definition, (2) provide protocol driven interventions to reduce rates of delirium (3) institute guidelines to more efficaciously treat those who become delirious (4) actively investigate delirious patient for signs of infection, perhaps even drawing blood cultures at the time of initial signs of delirium. A strength of this study was the individual chart reviews conducted on each of the septic patients, through which time lines were clearly delineated. Furthermore, this study included a very large cohort of patients.

It has previously been established in non-cardiac surgical and intensive care populations that delirium is associated with an increased risk for in-hospital morbidity, and poorer long-term outcomes. We have identified another adverse outcome associated with delirium: sepsis. Given the advancing age and increased medical co-morbidities of patients putting them at increased risk of developing in-hospital delirium, along with the increased focus on improving post-operative outcomes, attention must be paid to preventing and managing delirium. Those at risk need to be identified early, and those who become delirious must be appropriately managed which should include active surveillance for infectious complications.

## Conclusions

Delirium is strongly associated with sepsis, and through this study has been demonstrated to frequently precede the development of sepsis. The development of delirium in post-operative patients needs to be taken seriously and treated aggressively.

## Key messages

• Delirium is common after cardiac surgery.

• Delirium is associated with sepsis, and importantly has now been shown to precede sepsis in some cases.

• Delirious patients should be closely monitored for the development of other post-operative co-morbidities.

• Delirium is not a benign or self-limiting process.

## Abbreviations

BMI: body mass index; CABG: coronary artery bypass grafting; CHF: congestive heart failure; COPD: chronic obstructive pulmonary disease; CVD: cerebrovascular disease; EF: ejection fraction; ICU: intensive care unit; LOS: length of stay; MI: myocardial infarction; OR: odds ratio; PVD: Peripheral Vascular Disease; RF: renal failure; ROC: receiver operating characteristic; SIRS: Systemic Inflammatory Response Syndrome; STS: Society of Thoracic Surgeons.

## Competing interests

The authors declare that they have no competing interests.

## Authors' contributions

BJM designed the study, conducted the chart review, assisted with statistical analysis, and drafted the manuscript. KJB performed the statistical analysis and aided in revisions of the manuscript. RJFB and RCA contributed equally in achieving institutional ethics approval and co-senior authors on this study.

## Authors' information

BJM is an Alberta Heritage Foundation for Medical Research (AHFMR) Clinical Fellow at the University of Calgary. KJB is the senior statistical analyst for the division of Cardiac Surgery at Dalhousie University. RCA is the Rudy Falk Clinician-Scientist and Assistant Professor in Surgery and Physiology at the University of Manitoba. RJFB is an assistantociate professor of surgery at Dalhousie University.
